# Information Bottleneck Driven Deep Video Compression—IBOpenDVCW

**DOI:** 10.3390/e26100836

**Published:** 2024-09-30

**Authors:** Timor Leiderman, Yosef Ben Ezra

**Affiliations:** Faculty of Electrical Engineering, Holon Institute of Technology, 52 Golomb Str., P.O. Box 305, Holon 58102, Israel

**Keywords:** deep video compression, wavelets, information bottleneck, neural networks

## Abstract

Video compression remains a challenging task despite significant advancements in end-to-end optimized deep networks for video coding. This study, inspired by information bottleneck (IB) theory, introduces a novel approach that combines IB theory with wavelet transform. We perform a comprehensive analysis of information and mutual information across various mother wavelets and decomposition levels. Additionally, we replace the conventional average pooling layers with a discrete wavelet transform creating more advanced pooling methods to investigate their effects on information and mutual information. Our results demonstrate that the proposed model and training technique outperform existing state-of-the-art video compression methods, delivering competitive rate-distortion performance compared to the AVC/H.264 and HEVC/H.265 codecs.

## 1. Introduction

Data compression is the science of reducing the amount of data used to convey information. It is important to ensure that the signal or data stream compressed represents only the information transmitted [1]. There are two types of compression techniques: (1) Lossless compression and (2) Lossy compression. Lossless compression is a class of data compression algorithms that allows the perfect reconstruction of the original data from the compressed data. Lossy compression (irreversible compression) is the class of data encoding methods that uses inexact approximations and partial data discarding to represent the data. Recently, advanced compression algorithms have been invented that focus on reducing data redundancy through predictive techniques. The spatial redundancy is the dependence between neighbouring pixels in each video frame; the temporal redundancy is a given motion compensation or dependence between consecutive video frames. The encoder uses these redundancies to predict future pixels. A better prediction ability improves a residual error. Therefore, fewer bits are required, and compression efficiency is improved [2]. The standardized video compression algorithm used for video coding is mainly based on discrete cosine transform (DCT) coding and motion compensation Video coding formats include the following features. MPEG (Moving Pictures Expert Group), MPEG2 is the basis for the Advanced Television Systems Committee (ATSC), Digital Television Standard and the European Digital Broadcast (DVB) implemented for both standard and high-definition transmissions around the world. MPEG4 is a method of defining the compression of audio and visual (AV) digital data. It is usually used for web (streaming media), Compact Disk (CD) distribution, voice (telephone, videophone) and broadcast television applications. AVC (Advanced Video Coding) or MPEG4 part 10 is the most commonly used format for recording, compressing, and distributing video content. One of the additional features of the AVC is Multiview Video Coding (MVC), also known as MVC 3D. It is a stereoscopic video coding standard for video compression that efficiently encodes video sequences captured simultaneously from multiple camera angles in a single video stream. HEVC (High-Efficiency Video Coding), also known as MPEG-H Part 2, is a video compression standard designed as a successor to the AVC [3]. The most viable alternative to DCT for image compression is Wavelet technology. Wavelet transform methods are considered the successors of Fourier analysis based on self-similarity [4]. They are very efficient in signal processing for denoising, cross-correlation, and data compressions, such as MPEG-4 and JPEG2000. The Joint Photographic Experts Group developed the JPEG2000 compression standard based on discrete wavelet transform (DWT) Multiwavelets are a generalization of scalar wavelets. The fundamental concept underlying everything related to wavelet analysis is multiresolution. Scalar wavelets use one scaling function for the coarse approximation and one wavelet function for the fine detail. Multiwavelets use several scaling functions, combining wavelet functions into function vectors. However, multiwavelets have not yet been used for video compression [5]. Wavelets can provide both frequency and location information. Wavelets can be viewed as a burst of energy with the dominant frequency. When a wavelet transform is applied, a sequence is walked across a sample sequence in a process known as convolution. The wavelet moves one sample at a time, and the convolution product evolves for each position. This idea of moving a wavelet over the image and picking out details shows how wavelets can give both frequency and location information, which behave like a high pass filter and extract the high-frequency detail of the image. The image is also convolved with the complementary scaling function that removes the high frequencies. The product of these actions is a set of wavelet coefficients representing the fine detail of the image, on the one hand; on the other hand, the image from which the fine detail has been removed [6]. Video compression can be improved by using neural networks. Neural networks can be used for pre-processing, post-processing, optimization, and segmentation algorithms. There is a lack of studies regarding neural network applications for reducing spatial and temporal redundancy in video encoding [3]. Neural networks are a core component of machine learning, where computers are trained to perform tasks by analyzing large sets of examples. Deep learning (DL), a specialized subset of machine learning, involves multi-layered neural networks that mimic the way the human brain ’learns’ from vast amounts of data. In each layer of the neural network, DL algorithms perform a series of calculations, making predictions and adjusting progressively to improve the accuracy of the outcomes over time. While deep learning has become a powerful tool for image classification, its application to image and video compression has been more limited [7]. Neural networks have shown great promise in video analysis but have so far contributed only incremental improvements to video encoding [3,7,8]. As Joy et al. [9] point out, recent advancements in deep learning-based video compression techniques suggest a potential paradigm shift in the field. These techniques show considerable promise in overcoming the limitations of traditional methods, with neural networks offering the potential for significant improvements in both compression efficiency and visual quality. Mochurad’s study [10] further emphasizes the potential of machine learning for video compression by comparing convolutional neural networks (CNNs) with conventional codecs. The study underscores the strengths of machine learning-based approaches, while also addressing their current limitations, paving the way for optimized neural network algorithms in future video compression technologies. Additionally, wavelet-based algorithms have shown strong performance in video compression, although there is still room for improvement in their application, particularly when combined with deep learning techniques. The Information Bottleneck (IB) principle is a fundamental concept in information theory that provides a framework for understanding the trade-off between compression and relevance preservation [11]. The central idea is to extract the most relevant information from an input variable while minimizing the information retained, effectively creating a compressed data representation. This principle has numerous applications in various fields, including machine learning, data analysis, and signal processing. The IB principle suggests that an optimal compression of an input variable *X* can be achieved by finding a compact representation *T* that preserves the relevant information about a target variable Y while discarding the irrelevant details. This is accomplished by minimizing the mutual information between *X* and *T*, while maximizing the mutual information between *T* and *Y*. The resulting compressed representation T can be used for various tasks, such as classification, prediction, or data storage and transmission. While the IB principle provides a solid theoretical foundation for deep neural network-based compression, its practical application presents several challenges. One of the main difficulties is balancing the trade-off between compression and reconstruction accuracy, as the network must learn to discard irrelevant information while preserving the essential features necessary for high-quality reconstruction. Another challenge lies in the complex and often non-linear relationships between input data and the target objective. With their expressive power, deep neural networks can potentially learn representations that deviate from the optimal IB solution, leading to suboptimal compression performance. Developing effective regularization techniques and optimization methods to ensure the network adheres to the IB principle is an active area of research. Additionally, applying the IB principle to deep neural networks is complicated by the inherent difficulty in estimating and optimizing the mutual information between the input, the compressed representation, and the target variable. Researchers are exploring various techniques, such as variational approximations and adversarial training, to overcome these challenges and improve the effectiveness of IB-based deep compression methods. This research aims to develop wavelet-based deep neural networks, utilize an information bottleneck-driven approach for video compression, and implement this novel approach to improve the existing video compression rates. We hope these powerful compression methods will be used in future codecs and become part of the new standards. Experimental results show that estimating and compressing motion information using our neural network-based approach can significantly improve compression performance. We introduce an open-source Tensorflow 2 implementation of the DVC model with an optical flow-based discrete wavelet transform.

## 2. Wavelet Theory

Wavelet was introduced by Jean Morlet in 1982. The Continues Wavelet Transform (CWT) [12]

(1) $$\Psi_{s,\tau }=\frac{1}{\sqrt{\left|s\right|}}\Psi \left(\frac{t-\tau }{s}\right),\;\;s,\tau \in R,\;s\neq 0$$

The initial wavelet is called the mother wavelet. The mother wavelet is dilated with scale parameter s and is translated with parameter τ. Dilating wavelets makes it necessary to normalize the energy for different scales. This normalization is conducted by dividing the wavelet by $\frac{1}{\sqrt{\left|s\right|}}$ a continuous wavelet series set of scaling functions plus the wavelet function is defined by

(2) $$f\left(x\right)=\sum_{s}a_{r_{0},s}\phi \left(x\right)+\sum_{r=r_{0}}^{\infty }\sum_{s}b_{r,s}\psi_{r,s}\left(x\right)\;\;\;\;f\left(x\right)\in L^{2}\left(R\right),\;\;r\geq r_{0}$$

where $r_{0}$ is the scaling parameter and *s* is the shift parameter

(3) $$a_{r_{0},s}=\int f\left(x\right)\phi_{r_{0},s}\left(x\right)\;dx\;(\mathrm{set}\;\mathrm{of}\;\mathrm{scaling}\;\mathrm{functions})$$

(4) $$b_{r,s}=\int f\left(x\right)\psi_{r,s}\left(x\right)\;dx\;(\mathrm{set}\;\mathrm{of}\;\mathrm{wavelet}\;\mathrm{functions})$$

The function f(x) is defined as:

(5) $$f\left(x\right)=\left\{\begin{array}{cc} e^{x} & 0\leq x\leq 1\\ 0 & \mathrm{otherwise} \end{array}\right.$$

When x is not the continuous variable, we define the signal as M number of samples (discrete time signal) $s\left(n\right)\;\;\;n=0,1,\ldots ,M-1$ The Discrete Wavelet Transform (DWT) is almost always applied in the form of a filter bank:

(6) $$W_{\phi }\left(j_{0},k\right)=\frac{1}{\sqrt{M}}\sum_{n}s\left(n\right)\phi_{j_{0},k}\left(n\right)$$

where $\frac{1}{\sqrt{M}}$ is normalization. $s\left(n\right)$ is like $f\left(x\right)$, *j* is like *r* and *k* is like *s*.

(7) $$W_{\psi }\left(j,k\right)=\frac{1}{\sqrt{M}}\sum_{n}s\left(n\right)\psi_{j,k}\left(n\right)\;;\;\;\;\;j\geq j_{0}$$

$W_{\phi }\left(j_{0},k\right)$ is called the “Approximation” and $W_{\psi }\left(j,k\right)$ is called the “Details”. IDWT:

(8) $$s\left(n\right)=\frac{1}{\sqrt{M}}\sum_{k}W_{\phi }\left(j_{0},k\right)\phi_{j_{0},k}\left(n\right)+\frac{1}{\sqrt{M}}\sum_{j_{0}}^{\infty }\sum_{k}W_{\psi }\left(j,k\right)\psi_{j,k}\left(n\right)$$

For the 2D case for images, the DWT is applied in both horizontal and vertical directions. The approximation and detail coefficients are defined as follows

(9) $$\phi \left(n_{1},n_{2}\right)=\phi \left(n_{1}\right)\phi \left(n_{2}\right)-{Approximation}_{LPF,LPF}$$

(10) $$\psi^{H}\left(n_{1},n_{2}\right)=\psi \left(n_{1}\right)\phi \left(n_{2}\right)-\;{Horizontal}_{HPF,LPF\;}$$

(11) $$\psi^{V}\left(n_{1},n_{2}\right)=\phi \left(n_{1}\right)\psi \left(n_{2}\right)-{Vertical}_{LPF,HPF}$$

(12) $$\psi^{D}\left(n_{1},n_{2}\right)=\psi \left(n_{1}\right)\psi \left(n_{2}\right)-{Diagonal}_{HPF,HPF}\;$$

where ϕ is the “Approximation” function and ψ is the “Details” function. for horizontal, vertical, and diagonal directions. The DWT provides a hierarchical decomposition of the signal, making it a powerful tool for compression tasks by efficiently capturing multiscale information. Approximations and detailed coefficients for various wavelets are provided in Table 1.

## 3. Information Bottleneck (IB)

The Information Bottleneck (IB) method offers a powerful framework for understanding and optimizing how information flows through neural networks, particularly in tasks like video compression, where both efficiency and accuracy are crucial [11]. In the context of video compression, the IB framework seeks to reduce the dimensionality of data while retaining the most relevant information for reconstruction, thus optimizing both compression rates and video quality. The basic idea IB method can be formulated as follows: Given input variable X, and output variable Y, the goal is to find a compressed representation T that retains maximal information about Y while being minimal in size.

The IB method formulates this as an optimization problem:

(13) $${min}_{p\left(T|X\right)}\left[I\left(X;T\right)-\beta I\left(T;Y\right)\right]$$

where:

- I(X;T) represents the compression cost of the mutual information between X and T.
- I(T;Y) represents the relevance of the mutual information between T and Y.
- β is a Lagrange multiplier that balances compression and relevance.

Entropy is the uncertainty of a single random variable. We can define conditional entropy as the entropy of a random variable given another random variable. The reduction in uncertainty due to another random variable is called mutual information. For random variables *X* and *Y*, this reduction is given by [13]:

(14) $$I\left(X;Y\right)=H\left(X\right)-H\left(X|Y\right)=\sum_{x,y}p\left(x,y\right)log\frac{p(x,y)}{p(x)p(y)}$$

Mutual Information I(X;Y) measures the information shared between variables *X* and *Y*. In a deep neural network, each layer Ti represents a transformation of the input X through the network. Mutual information I(X;Ti) and I (Ti;Y) can be tracked across layers to understand the information flow in neural networks better. By balancing the trade-off between compression and relevance, neural networks can be guided to learn efficient and effective representations.

## 4. Proposed Method

**Figure 1:**
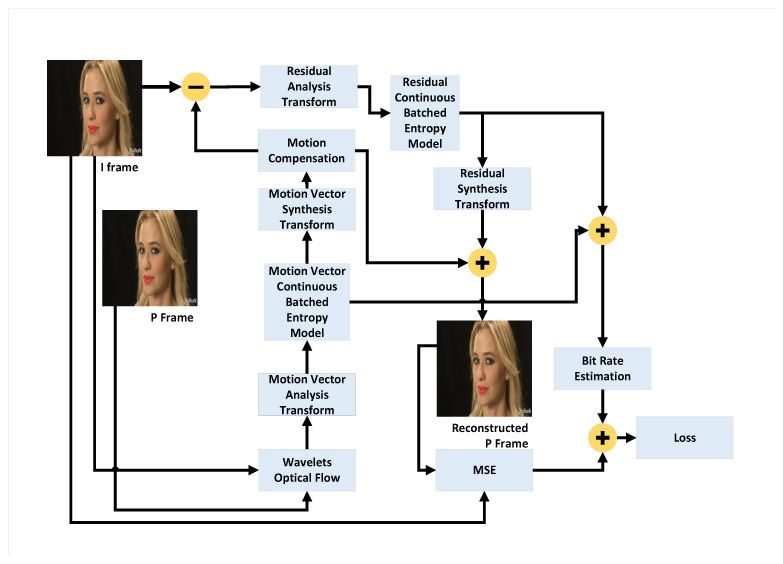
High-Level framework of the OpenDVCW Network.

**Figure 2:**
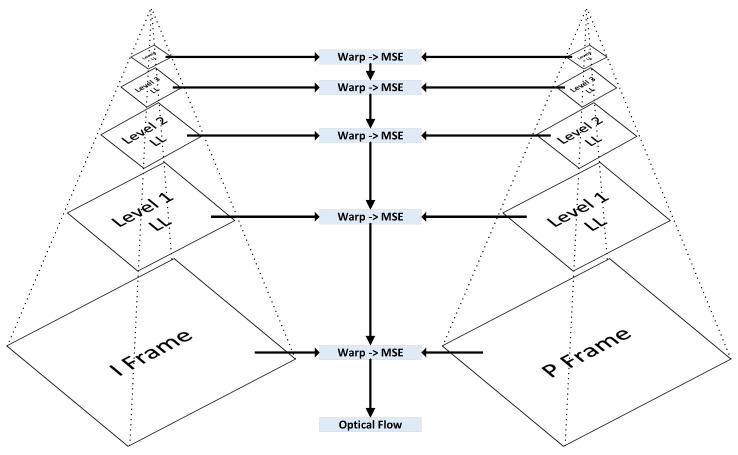
Pyramid architecture of the optical flow estimation.

**Figure 3:**
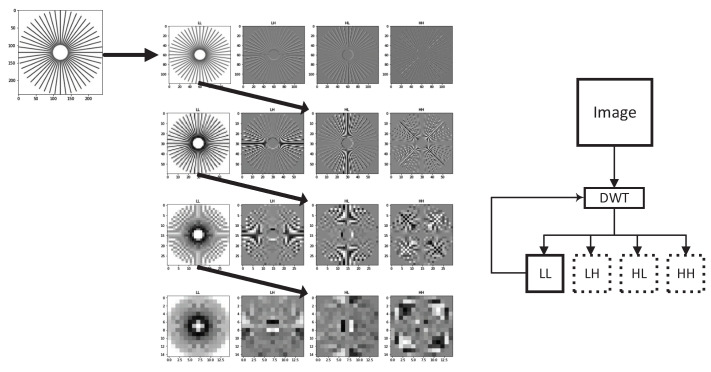
Visualization of DWT as we apply the transform on the approximation on every iteration.

This section presents the implementation of our Information Bottleneck-Driven, Wavelet-based OpenDVC-IBW model, which builds upon the OpenDVC framework [14,15]. Our enhancement replaces the traditional Average Pooling layers with Discrete Wavelet Transform (DWT) layers. Pooling layers play a critical role in deep neural networks (DNNs), providing benefits that enhance the performance and efficiency of neural networks, particularly in compression tasks. The proposed method also incorporates an analysis of information flow and mutual information within the neural network. An overview of the end-to-end video compression framework is shown in Figure 1. Like traditional video compression methods, our model follows the predict-transform architecture. The structure of the Motion Vector (MV) encoder network, the residual encoder network, and the motion compensation network follows the design of the OpenDVC architecture. However, we leverage wavelet properties to perform motion estimation directly on the wavelet coefficients. In the MV network, motion estimation is conducted using a pyramid structure, as shown in Figure 2, to estimate motion between consecutive frames. We employ a coarse-to-fine motion estimation strategy, which has been successful in previous works [16]. Following the settings in OpenDVC, we use a five-level pyramid network. However, instead of AveragePooling2D layers, we integrate DWT layers. For each pyramid level, we utilize the output of the DWT, where only the coarse estimation (i.e., approximations) is used, and the finer details are discarded, as illustrated in Figure 3. The DWT layers were implemented in TensorFlow 2, with the full implementation available at https://github.com/Timorleiderman/tensorflow-wavelets (accessed on 1 August 2024). Our TensorFlow 2 implementation of the model can be found at https://github.com/Timorleiderman/OpenDVCW (accessed on 1 August 2024). The model’s input consists of two frames: an I-frame (the reference frame) and a P-frame (the frame to be coded). The output is the latent binary representation of the coded P-frame, generated by the context-adaptive entropy coder. The decoder takes the I-frame and the binary representation of the P-frame as inputs to decode the P-frame.

## 5. Training Strategy

The OpenDVCW network was trained on the Vimeo90k. The whole network is jointly trained in an end-to-end manner using the loss of

(15) $$L=\lambda D\left(x_{t},\bar{x_{t}}\right)+R\left({\hat{m}}_{t}\right)+R\left({\hat{y}}_{t}\right)$$

The λ is a hyperparameter, also called the Lagrange multiplier, is used to balance the rate and distortion loss. D is the distortion defined as the mean square error (MSE), and R indicates the bit rate of the motion vectors and residual, respectively; the bit rate can be estimated as the cross entropy of the probability function. The probability function is calculated via a differentiable entropy model by adding a uniformly distributed noise with a mean of 0 and a width of 1. The learning rate was initially set to $10^{-4}$. Cosine decay was used to lower the learning rate as the training progressed.

## 6. Experiments and Results

**Figure 4:**
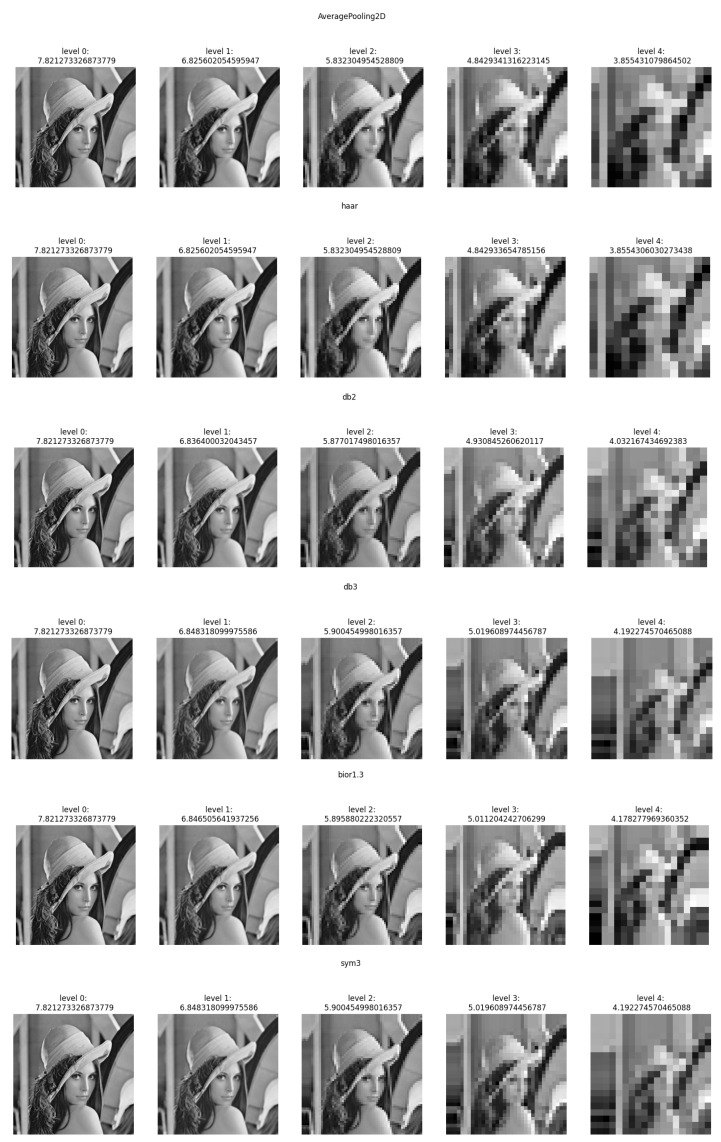
Calculated information on Lenna image for various mother wavelets.

The experiment had two primary objectives. First, we assessed the network’s information and mutual information [17] flow by employing various wavelets at the pooling layer. Second, we evaluated the quality and performance of the video coding. We investigated the dependence of an Average Peak signal-to-noise ratio (PSNR) vs. Bits Per Pixel (BPP) for various wavelets implemented at the pooling layer of the end-to-end video compression framework shown in Figure 1. In addition, we have compared the performance of the proposed method AVC/H.264, HEVC/H.265 and VVC/H.266 encoders. We have studied the influence of various wavelets on information and mutual information. The experiment was conducted on the Lenna image. First, we performed the discrete wavelet transform and retained only the approximation coefficients, setting all the detail coefficients to zero. Next, we applied the inverse discrete wavelet transform and calculated the mutual information between the original image and the reconstructed one. Figure 4 presents the results of the calculated information at different decomposition levels for various mother wavelets.

Table 1 presents the information for various wavelets at the different decomposition levels. The calculated information depends on the mother wavelet and the decomposition level. Column 1 and column 2 are exactly the same because of the averaging pooling and Haar decomposition equivalence. At the higher decomposition levels, information is higher for db3 and sym3 than other mother wavelets shown in Table 1. Table 2 presents the calculated mutual information for the various wavelets. The mutual information was calculated using the method described by Kraskov et al. (2004) [17].

**Figure 5:**
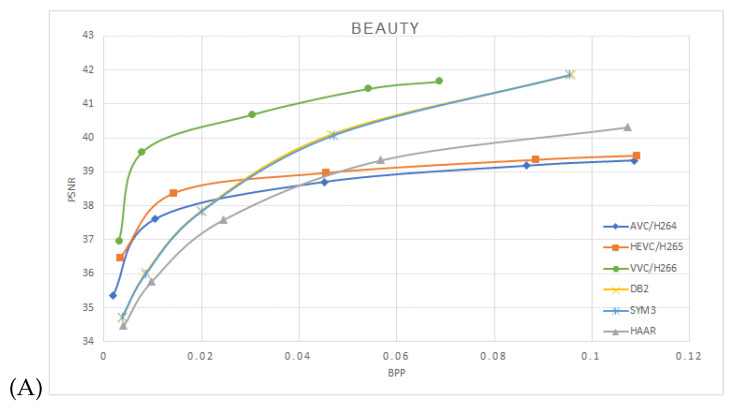

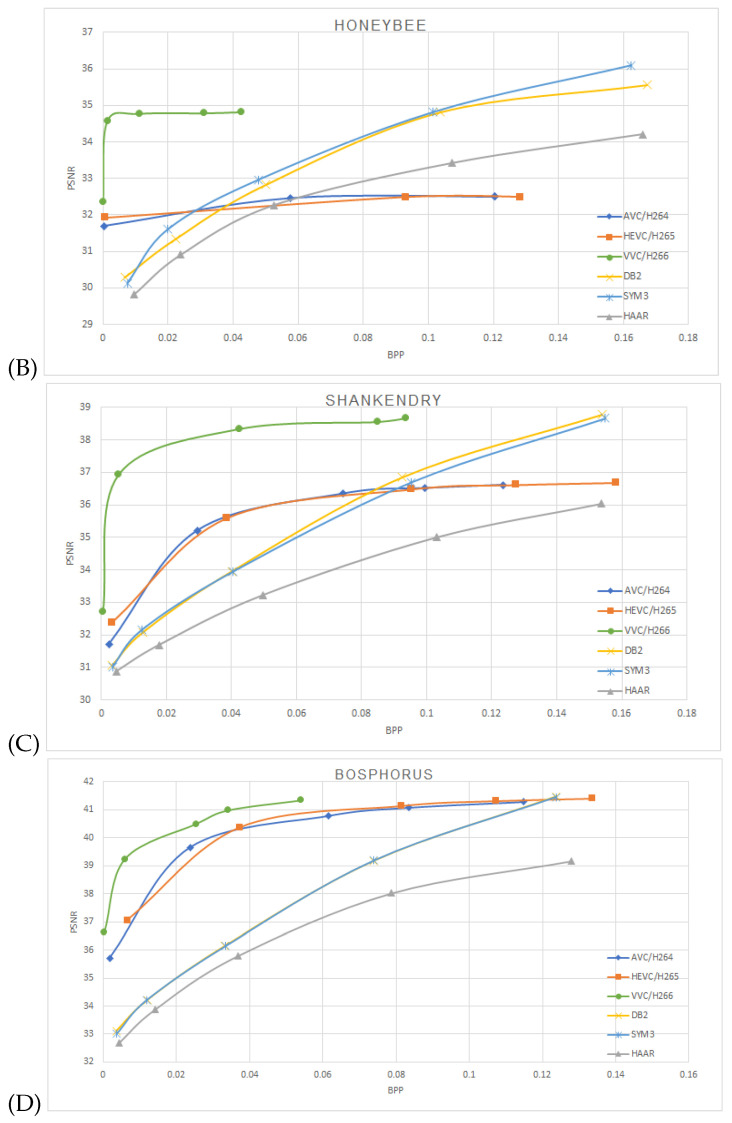
Performance on the UVG dataset comparison between AVC/H.264, HEVC/H.265, VVC/H.266 and OpenDVCW with Db2, Sym3 and Haar wavelets for the DWT transform in the optical flow. (**A**)—Beauty, (**B**)—HoneyBee, (**C**)—ShakeNDry, (**D**)—Bosphorus.

One can notice that the estimated mutual information between the network layers depends on the mother wavelets utilized at the pooling layer. The approximation coefficients used at the Haar wavelet’s layers are equivalent to averaging pooling layers. Utilizing db2 or sym3 wavelets increases the mutual information compared to the averaging pooling (Haar wavelet). We have used the Vimeo-90K dataset for the compression performance study to train our model. It consists of 89,800 video clips downloaded from vimeo.com, which cover various scenes and actions. It is designed for four video processing tasks: temporal frame interpolation, video denoising, video deblocking, and video super-resolution. BPG 444 [18] was used to compress I-frames in each video sequence with QP 22 27, 32, 37 and 42. The corresponding models were trained with lambda 65,536, 16,384 4096, 1024 and 256, respectively. A custom data generator was implemented to randomly sample two frames—“I-frame” and “P-frame” from the dataset. Then, the two frames were cropped to 240 × 240 resolution before they were fed into the model. We trained the network with mini-batches of 60,000 samples with 15 epochs. ADAM optimizer with cosine decay was used for the learning rate starting from $10^{-4}$. We then used Nvidia RTX 3060 with 12 GB GDDR6 to train the model, a process that lasted an average of about 24 h each. The proposed model was performed on the Open Ultra Video Group (UVG) dataset [19]. The UVG dataset is composed of 16 versatile 4K (3840 × 2160) test video sequences captured at 50/120 fps. The image sequence was resized to a 240 × 240 resolution to match the input size of the trained model. Compressed frames for AVC/H.264, HEVC/H.265, and VVC/H.266 were generated using libavcodec with libx264, libx265, and libvvenc, respectively. The encoders were configured with the “very fast” preset for AVC/H.264 and H.265, and the “faster” preset for H.266, consistent with the settings used in OpenDVC experiments. We trained the models HAAR-10k based on the Haar wavelet with 10,000 image pairs (I-Frame and P-Frame) with 15 epochs; HAAR-60k, DB2 (Daubechies 2) wavelet and SYM3 (Symlets 3) wavelet were trained with 60,000 image pairs. Figure 5 shows the average peak signal-to-noise ratio PSNR vs bits per pixel BPP on the encoded frames tested on the UVG dataset, A, B, C, and D are the video sequences Beauty, HoneyBee, ShakeNDry, and Bosphorous, respectively.

As shown in Figure 5, db2 and sym3 perform better than Haar, which is equal to average pooling, especially at higher BPPs. From Figure 5, it can be seen that for higher rate distortion ratios, our model achieves better performance compared to the conventional encoders.

## 7. Summary and Discussion

This paper proposes Information Bottleneck-Driven Deep Video Compression with Wavelet Transform. Our studies show that replacing the commonly used average pooling with wavelet transform-based pooling improves the performance of video compression rates. The improvement is particularly evident with wavelets, as they lead to higher mutual information between the layers. In terms of computational complexity, when using the HAAR wavelet, the complexity remains the same as in the original OpenDVC model, ensuring no additional computational overhead. However, using more complex wavelets can increase the computational cost. To balance performance and complexity, we opted to work with small filter taps like Daubechies (db2), (db3), and Symlet (sym3), which offer a good trade-off between accuracy and efficiency while keeping the computational complexity manageable. The proposed IBOpenDVCW network was carried out using TensorFlow 2. We have replaced the average pooling with DWT layers, implementing the pyramid algorithm for the optical flow estimation to obtain better quality and performance in video coding. The proposed network achieves equal or better results than the AVC/H.264, HEVC/H.265 and VVC/H.266 coders regarding BPP and PSNR. Increasing the dataset size and the number of epochs while training the model leads to better distortion rates. Future work may focus on developing and training the network with different kinds of wavelet families and researching and developing the network while using the discrete complex wavelet transform and accelerating the inference time. We have studied the mutual information between the layers for the different wavelets, as shown in Table 3. Additionally, the mutual information calculated for further wavelets is presented in Table 4.

We can see that for coif3 wavelets, mutual information is higher than for db2, db3, and sym3. Further study is required to check the compression rate improvement for these wavelets. It is not obvious that the wavelets with higher mutual information will improve because of the potential trade-off between mutual information and accuracy. In summary, pooling layers play a crucial role in deep neural networks by reducing dimensionality, aiding in translation invariance, preventing overfitting, and abstracting features. The Information Bottleneck theory provides a theoretical framework for balancing compression and information retention in image and video compression tasks. The choice of pooling method can significantly influence the performance of a network, with various techniques are available depending on the application’s specific needs.

## Figures and Tables

**Table 1:** Wavelet filter coefficients.

Wavelet_Name	LPF Filter	HPF Filter
haar	0.7071067811865476, 0.7071067811865476	−0.7071067811865476, 0.7071067811865476
db2	−0.12940952255126037, 0.2241438680420134, 0.8365163037378079, 0.48296291314453416	−0.48296291314453416, 0.8365163037378079, −0.2241438680420134, −0.12940952255126037
db3	0.03522629188570953, −0.08544127388202666, −0.13501102001025458, 0.45987750211849154, 0.8068915093110925, 0.33267055295008263	−0.33267055295008263, 0.8068915093110925, −0.45987750211849154, −0.13501102001025458, 0.08544127388202666, 0.03522629188570953
sym3	0.035226291882100656, −0.08544127388224149, −0.13501102001039084, 0.4598775021193313, 0.8068915093133388, 0.3326705529509569	−0.3326705529509569, 0.8068915093133388, −0.4598775021193313, −0.13501102001039084, 0.08544127388224149, 0.035226291882100656

**Table 2:** Entropy at each pyramid level of the decomposition for various mother wavelets.

	AveragePooling2D	DWT-haar	DWT-db2	DWT-db3	DWT-sym3	DWT-bior1.3
level 0	7.821273	7.821273	7.821273	7.821273	7.821273	7.821273
level 1	6.825602	6.825602	6.836400	6.848318	6.848318	6.846506
level 2	5.832305	5.832305	5.877017	5.900455	5.900455	5.895880
level 3	4.842934	4.842934	4.930845	5.019609	5.019609	5.011204
level 4	3.855431	3.855431	4.032167	4.192275	4.192275	4.178278

**Table 3:** Mutual Information for various mother wavelets.

Wavelet_Name	Mutual_Information
haar	2.894183
db2	3.159218
db3	3.215511
sym3	3.215256

**Table 4:** More results for Mutual Information for various mother wavelets.

	Wavelet_Name	Mutual_Information
1	coif2	3.267934
2	coif3	**3.282238**
3	sym2	3.159884
4	bior1.3	2.858263
5	bior2.2	3.270967
6	rbio1.3	3.246917
7	rbio2.2	2.962761

## Data Availability

Data is contained within the article.
